# Might E-cadherin promoter polymorphisms of rs16260 and rs5030625 associate with the risk of nephrolithiasis?

**DOI:** 10.1186/s40064-016-3363-2

**Published:** 2016-09-29

**Authors:** Cigdem Donmez, Ece Konac, Batuhan T. Aydogan, Cenk Y. Bilen

**Affiliations:** 1 Department of Medical Biology and Genetics, Faculty of Medicine, Gazi University, Besevler, 06510 Ankara, Turkey; 2 Department of Urology, Faculty of Medicine, Hacettepe University, Sıhhiye, 06100 Ankara, Turkey

**Keywords:** E-cadherin, Nephrolithiasis, Regulatory SNPs (rSNPs), Risk factors

## Abstract

**Purpose:**

To study whether −160 C > A (rs16260) and −347 G > GA (rs5030625) single nucleotide polymorphisms of the regulatory region (rSNPs) of *CDH1* gene modulate the risk of nephrolithiasis.

**Methods:**

Genomic DNA of 101 patients with calcium oxalate nephrolithiasis and 114 healthy controls were screened for both polymorphisms, using polymerase chain reaction-restriction fragments length polymorphism method (PCR-RLFP). Haplotype frequencies were also analyzed. To determine the association of rSNPs of *CDH1* gene with the clinicopathological features of nephrolithiasis, nearly all possible etiological factors were documented. These factors were family history, gender, age, body mass index, liquid consumption, eating habits, tea–coffee and meat (oxalate rich) consumption, adequate physical activity, and all serum and urine levels—the serum levels of Na, K, Cl, phosphate, Ca, Mg, uric acid, albumin, blood urea nitrogen (BUN), creatinine and serum parathyroid hormone (PTH) as well as 24 h urine excretions of creatinine, Na, K, Cl, phosphate, Ca, Mg, citrate, oxalate, uric acid, albumin and BUN.

**Results:**

Significant differences were found between rs16260 and the risk of nephrolithiasis. Patients having CA genotype of rs16260 *CDH1* polymorphism were associated with an almost trifold increased risk for developing kidney stone than those with the AA genotype (95 % CI 1.08–7.28, OR 2.8, *P* = 0.033). We also found that non-A allele carriers (CC) had significantly higher nephrolithiasis risk associated with the clinicopathological characteristics including serum calcium (*P* = 0.027) and 24 h urinary magnesium level (*P* = 0.042). Moreover, we did find a directly proportional relationship between the CA genotype and serum calcium levels (*P* = 0.041). There was no significant difference between patients and controls in terms of the distribution of rs5030625 genotypes and alleles (*P* > 0.05). Likewise, no associations between the rs16260 and rs5030625 haplotypes and susceptibility to kidney stone were observed (*P* > 0.05).

**Conclusion:**

Regulatory variants of rs16260 of the *CDH1* gene may confer susceptibility to nephrolithiasis. This may have important implications for understanding the pathophysiological mechanisms of the disease and suggesting novel targets for drug treatment.

## Background

Genetic and environmental factors greatly affect nephrolithiasis which is one of the most prevalent disorders observed in the urinary tract system (Amato et al. 2004). Single-candidate genes as well as epigenetic processes constitute the complicated genetic background of stone formation. Therefore, it is not easy to identify which gene plays a bigger role in stone formation.

The interaction of multiple genes leads to idiopathic calcium oxalate (CaOx) nephrolithiasis which is the most common form of urolithiasis. These genes can be classified under any one of the seven subgroups: receptors (vitamin D and calcium-sensing), ion channels (claudin 16 and 19), transporters (sodium-phosphate co-transporter), calcium channels (transient receptor potential cation channel subfamily V member 5 and 6), bicarbonate exchanger (soluble adenylate cyclase), chloride/H^+^ antiporter (CLCN5) and β-glucuronidase (klotho) (Monico and Milliner 2011). CaOx stones are the key elements in the most common form of renal stones and due to oxidative stress (OS), which is considered a major risk factor for crystallization and crystal deposition in kidneys, these stones have been reported to be injurious to the renal epithelial cells (Escobar et al. 2008). Loss of renal cell integrity due to damaging effect of CaOx crystals may be one of the reasons for development of kidney stones (Tsujihata 2008; Davalos et al. 2010; Thamilselvan et al. 2012).

Cadherins are transmembrane glycoproteins that mediate calcium-dependent cell–cell adhesion and regulate cellular behavior. E-cadherin (*CHD1*), one of the members of the cadherin protein family, plays an important role in maintaining epithelial integrity, cell polarity and tissue architecture. It is expressed in almost all epithelial cells including renal tubule cells (Wheelock and Johnson 2003; Takeichi 2004).

The studies on genetic polymorphisms, especially single nucleotide polymorphisms (SNPs), have attempted to identify genes associated with a variety of diseases. SNPs can be found in any region of the gene, structural or regulatory or even non-coding. The SNPs that have functional implications on the levels of gene expression are called regulatory SNPs (rSNPs), which change the transcriptional factor binding sites (TFBS). Therefore, functional rSNPs in TFBS may cause differences in gene expression, phenotypes and susceptibility to environmental exposure (Chorley et al. 2008).

SNPs in the *CHD1* gene might influence its expression by altering the level of gene transcription or translation and by affecting its messenger RNA stability. Previous studies (Tan et al. 2013; Tsai et al. 2003) have shown that SNPs in the *CHD1* gene might have altered the risk of nephrolithiasis development. This current study, for the first time as far as we are concerned, investigates the association of rSNPs [rs16260: −160 C > A; rs5030625: −347 G > GA (insertion mutation)] and the linkage disequilibrium analysis of these regions with the nephrolithiasis risk factors including clinical covariates in Turkish population.

## Methods

### Study subjects and blood samples

Our study group comprised 101 patients (aged 47.4 ± 15.1) with kidney stones who were treated at the Urology Department of the Hacettepe University. Stone samples were obtained either after extracorporeal shock wave lithotripsy or surgery, and all patients had stone analysis results confirming that their stone composition was calcium oxalate monohydrate or dihydrate. Excluding criteria of this study, for both patients and controls, consisted of any history of chronic urinary tract infection, renal failure, chronic diarrhea, gout, renal tubular acidosis, primary and secondary hyperparathyroidism and cancer. The control group included 114 subjects (aged 48.7 ± 14.6, gender matched to the patients) without a history of kidney stone or family history of stone diseases, all of which were recruited from the same area. The subjects who only had normal glomerular filtration rates (according to Cockroft–Gault formula) were included in the study. We asked all the study group participants to collect 24 h urine sample and sent the samples to the lab to obtain 24 h urine sodium (Na), potassium (K), chlor (Cl), phosphate, oxalate, citrate, calcium (Ca), magnesium (Mg), uric acid, albumin, blood urine nitrogen (BUN) and creatinine levels. Moreover, serum levels of Na, K, Cl, P, Ca, Mg, uric acid, albumin, BUN, creatinine and serum parathyroid hormone (PTH) were measured biochemically. We also looked into urine pH in morning urine sample. Individuals were interviewed to get information about their life style habits, namely physical activity, eating and water consumption. The study was approved by the ethical committee of the Gazi University, Faculty of Medicine. Informed consent was obtained from all subjects.

### Genotyping

5-ml blood sample was collected from the peripheral vein of each individual and was anticoagulated with EDTA. DNA was extracted from the blood sample using a Genomic DNA Extraction kit (Exgene™ Tissue SV, GeneAll Biotechnology, Seoul, Korea), according to the manufacturer’s instructions. We measured the concentration and purity of each sample DNA by using a Nanodrop spectrophotometer (NanoDrop® ND-1000, Thermo Scientific, USA). The ratio of the absorbance at 260 and 280 nm (A_260/280_) was used to assess the purity of DNA. For pure DNA, A260/280 is ~1.8. Amplifications of the −160 C > A (rs16260) and −347 G > GA (rs5030625) regions of the Cadherin-1 (*CDH1)* gene were carried out by placing in a Mastercycler gradient (Thermo Scientific, USA) thermal cycler, a total volume of 25 µl PCR mixture containing 100 ng genomic DNA, 1.5 μl of both forward and reverse primer (10 pmol/μl), 1 μl dNTP (10 mM), 1 μl 10X PCR buffer (with 15 mM MgCl_2_), 0.4 μl 2 units *Taq* DNA polymerase. The primers were synthesized by NZYTech, Lda (Lisboa, Portugal). For each SNP, we used the following set of primers: rs16260-forward 5′-TCCCAGGTCTTAGTGAGCCA-3′ and rs16260-reverse 5′-GGCCACAGCCAATCAGCA-3′, rs5030625-forward 5ʹ-GCCCCGACTTGTCTCTCTAC-3ʹ and rs5030625-reverse 5ʹ-GGCCACAGCCAATCAGCA-3ʹ. The following PCR procedure was used: initial denaturation at 95 °C for 5 min; 35 cycles of denaturation at 95 °C for 30 s; annealing at 60 °C for 40 s and extension at 72 °C for 45 s; and a final elongation at 72 °C for 10 min. Following PCR, 5 μl amplified products were digested overnight with 5 units *Hph*I (New England Biolabs, Ipswich, MA) for −160 C > A (rs16260) at 37 °C and with 5 units *Ban*II (New England Biolabs, Ipswich, MA) for −347 G > GA (rs5030625) restriction endonucleases at 37 °C. For the −160 C > A nucleotide substitution in promotor of *CDH1* gene, −160 C allele was cut into two fragments of 100 and 90 bp while the −160 A allele remained uncut (190 bp). For the −347 G > GA (insertion mutation) in promotor of *CDH1* gene, the GA alleles were represented by DNA bands of sizes 332 and 116 bp and the G alleles were represented by DNA bands of sizes 263, 116 and 68 bp, whereas the heterozygotes displayed a combination of both alleles (332, 263, 116 and 68 bp). Following digestion, the DNA fragments were separated by electrophoresis for 1 h at 100 V on an agarose gel containing GelRed™ Nucleic Acid Gel Stain (Biotium, Hayward, CA, USA). After the incubation time, the fragments were separated on a 3 % agarose gel that was stained with GelRed™ and visualized by Gel Logic 100 image system (Kodak, Rochester, NY). Genotype analysis was performed by two persons independently in a blind fashion. About 10 % of the samples were randomly selected for repeated genotyping. The results were 100 % concordant.

### Statistical analysis

Tests for Hardy–Weinberg equilibrium, which revealed normal distribution of the population, were performed by the Chi square (χ2) test. Then, the differences in genotype distribution and allele frequencies among the groups were examined for statistical significance using the Pearson’s two-way Chi square (χ2)-test. When the assumption of the χ2 failed, Fisher’s exact test was performed. The relationships between genotypes and/or alleles and risk of nephrolithiasis were determined by obtaining the Odds Ratios (ORs) through a logistic regression method [OR 95 % Confidence Interval (CI)]. Adjusted ORs for the other clinical covariates including family history, body mass index, liquid consumption, eating habits, physical activity, serum and urine values were determined by using multivariable logistic regression. *P* values smaller than 0.05 were considered statistically significant. All analyses were conducted by using R statistical programming language, also called GNU S, v. 2.15.0.

## Results

There were no differences between patients and controls in terms of gender and age distribution and body mass index (BMI) (*P* > 0.05). Eating habits, including daily salt consumption, water intake, tea–coffee and meat (oxalate rich) consumption were also found similar between the two groups. The genotype frequencies of both rSNPs were in agreement with Hardy–Weinberg equilibrium among the patients and controls (*P* > 0.05). Genotype and allele frequencies of −160 C > A (rs16260) and −347 G > GA (rs5030625) *CDH1* polymorphisms and their association with the risk of nephrolithiasis are shown in Table 1. Out of 101 patients, 10 cases were type AA, 39 cases were type CA and 52 cases were type CC. No significant differences in genotype (*P* = 0.124) and allele (*P* = 0.227) frequencies for rs16260 polymorphism were observed between patients and controls. ORs were calculated by comparing the AA homozygote genotype with the others variant genotypes, CA, CC, CA+CC. When compared to the AA genotype, the OR for nephrolithiasis risk of patients with CA and CA+CC genotypes were found statistically significant (*P* = 0.033) and at the very edge of significance (*P* = 0.053) respectively (Table 1). With regard to the genotype distribution for rs5030625 *CDH1* polymorphism in patients, 8 cases were type GA/GA, 25 cases were type G/GA and 68 cases were type GG. There were no significant differences in rs5030625 polymorphism between the patient and control groups (*P* > 0.05). Logistic regression analysis was run for each genotype to estimate the risk for developing nephrolithiasis. None of the allele types (GA or G) for each genotype was found to be a significant risk factor (Table 1).

**Table 1:** Genotype and allele frequencies of *CDH1* promotor polymorphisms in patients with nephrolithasis and controls

Genotypes and alleles	Patients (n = 101) n (%)	Controls (n = 114) n (%)	P values*	OR (95 % CI)*	P values**
HphI (rs16260, −160 C > A)			0.124		
AA	10 (9.9)	22 (19.2)		1	
CA	39 (38.6)	35 (30.7)		2.80 (1.08–7.28)	*0.033*
CC	52 (51.4)	57 (50.0)		2.16 (0.87–5.33)	0.094
CA + CC	91 (90)	92 (80.7)		2.17 (0.97–4.85)	0.053
Alleles			0.227		
A	59 (29.2)	79 (34.6)		1	
C	143 (70.7)	149 (65.3)		1.28 (0.85–1.93)	0.2281
*Ban*II (rs5030625, −347 G > GA)			0.523		
GA/GA	8 (7.92)	5 (4.38)		1	
G/GA	25 (24.7)	27 (23.6)		0.53 (0.14–2.05)	0.359
G/G	68 (67.3)	82 (71.9)		0.45 (0.13–1.60)	0.220
G/G + G/GA	93 (92)	109 (95.5)		0.53 (0.17–0.69)	0.278
Alleles			0.333		
GA	41 (20.2)	37 (16.2)		1	
G	161 (79.7)	191 (83.7)		0.76 (0.46–1.24)	0.275

Italics values means P value < 0.05

*OR* odds ratio, *95* *% CI* 95 % confidence interval

* *χ* ^2^ analysis between patients and controls

** P values were calculated AA vs. CA, CC and CA+CC; GA/GA vs. G/GA, G/G and G/G+G/GA by χ^2^ test

Four haplotypes were revealed after the combination of *CDH1* polymorphisms, HphI and* Ban*II, which were in high linkage disequilibrium. When the putative risk genotypes were combined and used as the reference A GA haplotype, a significant difference was not seen for any subject groups (*P* > 0.05) (Table 2).

**Table 2:** HphI (rs16260) and* Ban*II (rs5030625) haplotype frequencies and risk estimates

Haplotypes	Patients (%) (2n = 202)	Controls (%) (2n = 228)	OR (95 % CI)	P values
A GA	12 (5.94)	11 (4.82)	1^a^	
A G	47 (23.26)	68 (29.82)	0.63 (0.25–1.55)	0.320
C GA	29 (14.35)	26 (11.4)	1.02 (0.38–2.70)	0.964
C G	114 (56.43)	123(53.94)	0.84 (0.36–2.00)	0.709

*OR* odds ratio, *CI* confidence interval

^a^ Reference haplotype

We measured, in both groups, the serum levels of Na, K, Cl, phosphate, Ca, Mg, uric acid, albumin, blood urea nitrogen (BUN), creatinine and serum PTH as well as 24 h urine excretions of creatinine, Na, K, Cl, phosphate, Ca, Mg, uric acid, albumin and BUN. Urine pH was similar between patients (6.2 ± 0.6) and controls (6.0 ± 0.4). There were almost no significant differences between patients and controls in terms of these biochemical covariant factors (data not shown) (*P* > 0.05), except for the serum calcium, 24 h urinary magnesium, citrate and oxalate levels. Therefore, in the rest of our analysis, we focused only on the relationship between rs16260 genotypes and these four biochemical parameters. We observed a significant effect modification only in levels of serum calcium and 24 h urinary magnesium in rs16260 (Table 3). Significant differences were found for CA (*P* = 0.041) and CC (*P* = 0.027) genotypes when the serum calcium levels in all three genotypes were compared individually. For the CA and CC genotypes, serum calcium levels were positively related to the risk of nephrolithiasis. There was a statistically significant association between the CC genotype patients and 24 h urinary magnesium level. In patients with the CC genotypes in comparison to the CA and AA genotypes, the adjusted ORs were found to be 3.43 (95 % CI 0.95–5.62, *P* = 0.042) for 24 h urinary magnesium levels (Table 3). For the CC genotype, 24 h urine Mg levels were found nearly 3.5 times lower than those for the AA genotype. In other words, for the CC genotype, 24 h urine Mg levels were inversely proportional to the risk of nephrolithiasis. Multivariable logistic regression analysis demonstrated that the occurrence of nephrolithiasis was not associated with 24 h urinary citrate (CA: *P* = 0.662 and CC: *P* = 0.136) or 24 h urinary oxalate (CA: *P* = 0.828 and CC: *P* = 0.670).

**Table 3:** Odds ratios (ORs) of −160 C > A (rs16260) of *CDH1* gene in nephrolithiasis patients with adjustments

*rs16260,* −*160 C* > *A*	OR^a, e^ (95 % CI)	P values^a, e^	OR^b, e^ (95 % CI)	P values^b, e^	OR^c, e^ (95 % CI)	P values^c, e^	OR^d, e^ (95 % CI)	P values^d, e^
AA	1		1		1		1	
CA	3.40 (0.95–4.97)	*0.041*	0.17 (0.39–1.98)	0.675	0.19 (0.35–1.94)	0.662	0.04 (0.38–2.13)	0.828
CC	4.86 (0.17–0.90)	*0.027*	3.43 (0.95–5.62)	*0.042*	2.21 (0.81–4.45)	0.136	0.18 (0.51–2.77)	0.670

Italics values means P value <0.05

*OR* odds ratio, *95* *% CI* 95 % confidence interval

^a^ OR adjusted for serum calcium level

^b^ OR adjusted for 24 h urinary magnesium level

^c^ OR adjusted for 24 h urinary citrate level

^d^ OR adjusted for 24 h urinary oxalate level

^e^ Calculations were performed AA vs. CA and CC

## Discussion

Nephrolithiasis remains one of the most common urological diseases in developing countries. The molecular events leading to nephrolithiasis are extremely complex. Although the explicit mechanism lying underneath the pathogenesis of stone formation has not been yet clearly defined, calcium and transforming growth factor-β1 (TGF-β1) have been thought to participate in the process.

The transmembrane protein CHD1 mediated adherens junction calcium-dependent cell adhesion and there is good evidence that the presence of E-cadherin is critical for epithelial development in the early mammalian embryo (Ojakian et al. 2001). Renal epithelial cells were shown to deteriorate after exposure to high levels of oxalate and CaO_x_ crystals (Ouyang et al. 2011; Khaskhali et al. 2009). The integrity of cell polarity and cell–cell adhesions (mainly E-cadherin-mediated adherens junction) were altered in the renal epithelial cells during nephrolithiasis. He et al. (2015) have recently reported that, pathogenesis of calcium stone development may have been associated with synergic effects of TGF-β1 and Ca^2+^, which promoted epithelial to mesenchymal transition (EMT) and osteochondral differentiation via Wnt11 and the L-type calcium channel. mRNA instability and down-regulation of the gene in mesenchymal tumor cells were shown to occur as a result of the regulatory sequence of *CHD1* gene. Many non-coding SNPs (regulatory SNPs) especially those that are in the vicinity of protein coding genes (promotor region) play important roles in shaping chromatin structure, regulate gene expression and, as such, are implicated in a wide variety of diseases. Thus *CHD1* rSNPs in conjunction with renal cell injury, caused by oxalate crystals, may contribute to the development of nephrolithiasis.

−160 C > A (rs16260) has been extensively scrutinized for its association with gastric (Chen et al. 2011), breast (Tipirisetti et al. 2013), colorectal (Pittman et al. 2009) and papillary thyroid (Wang et al. 2012) cancers and several noncancerous diseases (Li et al. 2014). Cloning the two alleles into the upstream of a promoterless luciferase reporter gene revealed that the A allele decreases transcriptional activity by 68 % compared with the C allele in a reporter gene analysis. This suggested that the A allele might have reduced E-cadherin expression in vivo (Li et al. 2000). While most studies on the −160C > A SNP focused on cancer, only one study has examined its association with noncancerous diseases including urolithiasis. In a hospital-based case–control study of 127 nephrolithiasis patients and 152 controls, Tan et al. (2013) genotyped the −160C > A SNP and found that CA/AA genotypes were associated with a significantly decreased risk of nephrolithiasis (OR 0.53; 95 % CI 0.32–0.87), compared with the CC genotype. Our findings regarding the distribution of this polymorphism were consistent with those of Tan et al. (2013), except for the CA genotype. Even though AA genotype was associated with a significantly decreased risk of nephrolithiasis in our population, CA variants were found to increase this risk. CA genotype of rs16260 *CDH1* polymorphism has increased stone formation risk almost threefolds compared with the AA genotype (95 % CI 1.08–7.28, OR 2.8, *P* = 0.033). This discrepancy might have resulted from ethnic composition differences between the studies. Other factors such as selection bias of subjects and different matching criteria may also have played their roles. In brief, alleles occur at different frequencies in different human populations as populations that are more geographically and ancestrally remote tend to differ more in terms of allele frequencies. We found CA+CC genotypes at the very edge of significance (*P* = 0.053). Overall, these results suggested that “A” allele may confer protective effects against nephrolithiasis. We found no significant differences in the allele frequencies between the patients and healthy control subjects. It is not clear why CA carriers of −160 C > A were likely to develop kidney stone. This could be because the possible binding sites of transcription factors may have been affected by the −160 C > A substitution of the promoter region. The C allele might have caused weak transcription factor binding affinity and low transcriptional activity in the expression of E-cadherin—a homophilic cell adhesion molecule which plays an important role in maintaining epithelial integrity due to its anti-invasive properties in numerous epithelial-derived cancers. Each of the two regulatory variant alleles in the hybrid cross has regulated expression of the other. We found that when stratified by serum calcium levels, the C allele carriers (CA and CC genotypes) had a significantly increased risk of nephrolithiasis. Significantly decreased 24 h urinary magnesium levels associated with increased nephrolithiasis risk were found in non-A allele carriers (CC). We confirmed that the A variant allele was likely to be protective against the development of nephrolithiasis. Magnesium inhibits calcium oxalate crystallization in human urine and may be highly useful as antilithiasis therapy (Grases et al. 2015).

Recent genome-wide research studies have showed that rSNPs affected transcription factor binding and chromatin structure, thus modulated neighboring gene expression (Kilpinen et al. 2013; Waszak et al. 2015). Other studies have pointed out that E-cadherin −160 C > A rSNP could modify the risk of several diseases. In this context, another SNP in the E-cadherin promoter, namely −347G > GA (rs5030625), which is also capable of modifying promoter transcriptional activity and disease risk has been identified (Shin et al. 2004). In our study, there were no significant differences between patients and controls in terms of the distribution of rs5030625 genotypes and alleles (*P* > 0.05). Likewise, no association between the rs16260 and rs5030625 haplotypes and susceptibility to nephrolithiasis was observed (*P* > 0.05). Chien et al. (2011) showed that individuals with the CDH1-347G/GA or GA/GA polymorphic genotypes had a significantly higher risk of hepatocellular carcinoma than those with the wild-type (G/G) genotype. However, the association of rs5030625 (CDH1-347G > GA) regulatory polymorphism with noncancerous nephrolithiasis has not been investigated in any known studies, thus we were unable to compare our results with those of similar studies covering other ethnic populations.

## Conclusion

Our study is the first to put forward the association between *CHD1* rSNP (rs16260) and nephrolithiasis by seeking linkages among the risky genotypes (CA and CC) and serum calcium and 24 h urinary magnesium levels. Further large population-based genome-wide association studies (GWAS) are necessary to clarify the potential function of *CHD1* rs16260 in the progression of nephrolithiasis. This is important especially for demonstrating polymorphic variants conferring modest relative risks.
